# The Human Epidermal Basement Membrane: A Shaped and Cell Instructive Platform That Aging Slowly Alters

**DOI:** 10.3390/biom10121607

**Published:** 2020-11-27

**Authors:** Eva Roig-Rosello, Patricia Rousselle

**Affiliations:** 1 Laboratoire de Biologie Tissulaire et Ingénierie Thérapeutique, UMR 5305, CNRS-Université Lyon 1, SFR BioSciences Gerland-Lyon Sud, 7 Passage du Vercors, 69367 Lyon, France; eva.roig-rosello@ibcp.fr; 2 Roger Gallet SAS, 4 rue Euler, 75008 Paris, France

**Keywords:** extracellular matrix, basement membrane, dermal-epidermal junction, skin, epidermal rete-ridge, dermal papilla, aging, mechanical properties

## Abstract

One of the most important functions of skin is to act as a protective barrier. To fulfill this role, the structural integrity of the skin depends on the dermal-epidermal junction—a complex network of extracellular matrix macromolecules that connect the outer epidermal layer to the underlying dermis. This junction provides both a structural support to keratinocytes and a specific niche that mediates signals influencing their behavior. It displays a distinctive microarchitecture characterized by an undulating pattern, strengthening dermal-epidermal connectivity and crosstalk. The optimal stiffness arising from the overall molecular organization, together with characteristic anchoring complexes, keeps the dermis and epidermis layers extremely well connected and capable of proper epidermal renewal and regeneration. Due to intrinsic and extrinsic factors, a large number of structural and biological changes accompany skin aging. These changes progressively weaken the dermal–epidermal junction substructure and affect its functions, contributing to the gradual decline in overall skin physiology. Most changes involve reduced turnover or altered enzymatic or non-enzymatic post-translational modifications, compromising the mechanical properties of matrix components and cells. This review combines recent and older data on organization of the dermal-epidermal junction, its mechanical properties and role in mechanotransduction, its involvement in regeneration, and its fate during the aging process.

## 1. Introduction

One of the most important functions of skin is to act as a protective barrier. To fulfill this role, the structural integrity of the skin depends on the basement membrane—a complex network of extracellular matrix (ECM) macromolecules that connect the outer epidermal layer to the underlying dermis. The epidermis, which consists primarily of keratinocytes, is continuously renewed by the proliferation of stem cells and the differentiation of their progeny. These cells undergo terminal differentiation as they exit the basal layer and move toward the surface, where they die and slough off [1]. Basal keratinocytes adjoin the dermal–epidermal junction (DEJ), a cell surface-associated ECM that acts in concerted action with both epidermal and dermal cells [2]. The DEJ provides both structural support to keratinocytes and a specific niche that mediates signals, influencing their behavior.

Much progress has been made in understanding the molecular structure and functions of extracellular macromolecules at the DEJ. Similar to all archetypal thin planar basement membranes, the DEJ consists primarily of laminins, collagen IV, nidogens, and the heparan sulfate (HS) proteoglycan perlecan, all of which are necessary for tissue organization and structural integrity [3,4,5]. Electron microscopy evaluation of the DEJ following conventional fixation protocols has revealed a lamina densa and lamina lucida, much like those of other basement membranes. In addition, the DEJ contains numerous regular structures known as anchoring complexes [6,7]. These anchoring complexes consist of electron-dense thickenings of the basolateral plasma membrane, which are called hemidesmosomes because of their resemblance to half of the desmosome plaques present on the plasma membranes at sites of cell-cell contact. Cytoskeletal keratin filaments insert into the hemidesmosomes, bridging the plasma membranes and providing a continuous intercellular network throughout the entire epidermis. Thin filaments known as anchoring filaments appear to traverse the lamina lucida and insert into the lamina densa, tethering the hemidesmosomes to the basement membrane [8,9]. Other structures, the anchoring fibrils, originate within the lamina densa and project into the upper regions of the papillary dermis. They either loop back and reinsert into the lamina densa or extend perpendicularly from the basement membrane and insert into anchoring plaques. Anchoring plaques are electron-dense condensations of the ends of anchoring fibrils together with other intrinsic constituents of the basal lamina [10,11]. Additional anchoring fibrils originate in the plaques and extend further into the papillary dermis. The complexity of these structures probably serves to increase the frictional resistance of external epithelia to applied forces [12].

In addition, the DEJ provides a highly dynamic microenvironment to the keratinocytes by participating in epidermal renewal under physiological conditions and taking part in repair processes during skin healing [13,14,15,16]. In both scenarios, the DEJ serves as an adhesive scaffold, and its constituents may be part of signaling events that vary depending on maturation processes driven by ECM-modifying enzymes [17,18]. Basement membranes can function as signaling platforms by sequestering growth factors and other ligands. Perlecan, agrin, and collagen XVIII bind to many growth factors via HS glycosaminoglycan chains [19,20]. Through binding and sequestering of soluble growth factors in the presence of appropriate cell-mediated forces or proteolytic degradation, basement membranes can also enable spatial-temporal regulation of receptor–ligand interactions. Furthermore, these ECMs can generate and transduce mechanical signals. Through interactions with cell surface receptors, these ECMs modulate a remarkably wide range of signaling processes.

## 2. General Organization of the Interfollicular DEJ

The originally inferred thickness of the DEJ, around 100 nm, was based on measurements made on transmission electron microscopy images taken of human skin cross-sections. The development of imaging approaches that require less or no chemical treatment of samples, such as atomic force microscopy (AFM) and super-resolution light microscopy, consistently have allowed re-evaluation of basement membrane thickness values, yielding results that double those obtained by transmission electron microscopy [5,21]. 

### 2.1. The DEJ Undulating Pattern and the Rete Ridges

**Figure 1:**
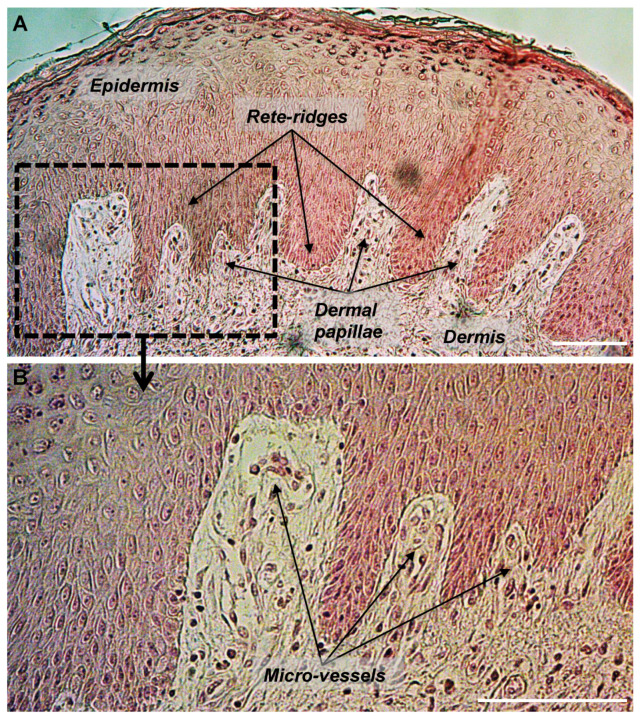
Histological section of human skin showing the undulated structure of the dermal-epidermal junction with epidermal rete-ridges and dermal papillae. Section of a paraffin-embedded skin biopsy from the abdomen of a 30 years old donor was processed for HES staining. (**A**) Epidermis, dermis, rete-ridges, and dermal papillae are shown. (**B**) Micro-vessels are shown within dermal papillae. Scale bars = 100 μm.

The DEJ displays a distinctive microarchitecture characterized by an undulating pattern arising from epidermal rete ridges that are downgrowths of the epidermis within the papillary dermis, leading to the characteristic varying number of strata along the epidermis [22] (Figure 1). These nipple-like elevations indent the epidermis layer and substantially increase the surface area of the DEJ, strengthening dermal-epidermal connectivity and keeping the dermis and the epidermis layer well connected. In addition, they increase the overall number of hemidesmosomes at the DEJ and subsequently improve the strength of the interface and of the mechanical properties of skin [23,24].

Rete ridges surround the dermal papillae, which can be seen as small extensions protruding from the papillary dermis within the epidermis. These two structures are perfectly embedded; the alternation of a rete ridge with a dermal papilla creates a characteristic repetitive and wave-shaped pattern. The macroscopic pattern of ridges and furrows that can be viewed on the external surface of the skin give rise to fingerprints. Furthermore, because the epidermis does not contain any blood vessels, blood supply, and flow of nutrients to the epidermis comes from capillaries within the dermal papillae. Each dermal papilla is endowed with at least one capillary loop [25]. 

Rete ridges in normal human skin vary based on anatomic location and donor age, with reported ranges of 50–400 μm in width and 50–200 μm in-depth [26,27]. Rete ridges tend to be shorter and less abundant in photo-exposed zones such as the forearm than in photo-protected zones such as the buttock [28] (Table 1). 

Of note, darker skin contains larger, more densely packed epidermal rete ridges as compared with lighter skin, regardless of age [35]. A study using high-resolution optical coherence tomography revealed that the average dermal papillae density of healthy human forehead skin is approximately 82 per mm^2^ [36]. A successful approach based on segmenting the DEJ by in vivo reflectance confocal microscopy using a three-dimensional conditional random field model was recently developed to quantify the modifications the DEJ undergoes during human skin aging in a more sensitive and specific manner [37]. Skin in areas of the body that experience excessive friction or shear stress, including the soles of the feet and the palms of the hands, exhibit more numerous, narrower, and deeper rete ridges than regions of lower friction such as the scalp. Studies of the oral mucosa have suggested that the morphogenesis of the rete ridge could result from physical forces that mechanically activate extracellular regulated protein kinase 1/2 (ERK1/2) to promote the proliferation and migration of the keratinocytes [38]. This association suggests that the length of the rete ridge is positively correlated with the strength of mechanical stresses. Reinforcing this hypothesis are results of a study showing that mechanical stretching of the skin in a porcine model induced integrin β1 subunit upregulation in keratinocytes, increased proliferation of the basal layer, and increased the number and height of epidermal rete ridges [39]. Of interest, multiscale mechanical characterization of human foot skin, tested using computational models of load bearing, demonstrated that the enhanced resistance of plantar skin to deformation and stress-induced injuries is linked to its dermal and epidermal layer composition rather than to its greater interdigitation pattern [40]. This shows that all cutaneous compartments are involved in the overall high resistance of the plantar tissue to deformation.

The rete ridges develop during mid-gestation, before which the DEJ is flat [41,42,43]. The formation of dermal ridges starts at approximately 10 weeks post-fertilization, when small-amplitude undulations of the basal laminae appear because of localized cellular proliferations in the epidermal basal layer [44]. The height of the rete ridges declines during skin aging [32,45,46,47,48], but it increases when the skin is hyperproliferative, as in psoriatic lesions [49,50,51].

It is unclear where interfollicular stem cells are located, in the undulating human basal compartment, in rete ridges, over dermal papillae, or all three. Some reports favor a protective location at the base of rete ridges [52,53,54], whereas other findings suggest a location over the tops of dermal papillae or troughs of the rete ridges where the epidermal basal layer comes closest to the skin surface [55,56,57]. Still, other studies report that epidermal stem cells are located along the basal layer and reside in both the rete ridges and over the dermal papillae, and do not seem to cluster at any specific location in the basal compartment [58].

### 2.2. Expected Impact of Rete Ridges in Epidermal Regeneration

Rete ridges are not reconstituted after full thickness wound healing in humans and most other mammals. In severe recessive dystrophic epidermolysis bullosa (EB)-affected skin in which the anchoring fibrils component collagen VII is defective, rete ridges are normal before epidermal blister formation but are affected during the resolution of the blister, and hyperkeratosis with scarring reduces rete ridges [59]. A flattening of the DEJ is also observed in cheloids, pathologic scars defined as fibroproliferative diseases resulting from abnormal wound responses, which grow beyond the original wound margins [60]. Interesting studies, however, have reported rete ridge regeneration after full or partial-thickness wounding in a few animal models, such as the Red Duroc and Lanyu pigs [61,62,63]. Wound healing of partial-thickness wounds in the Lanyu pig involves an alkaline phosphatase-positive cell population present at the bottom of the rete ridge basal layer [63]. Because of their importance in epidermal homeostasis, rete ridges may have a role to play in the process of skin repair. Epidermal regeneration describes the resurfacing of a skin wound with a new functional epidermis. This step depends on reconstitution of the DEJ, which anchors the epidermis to the dermis, and on the terminal differentiation of keratinocytes into a protective cornified layer [64]. The absence of rete ridges during this process after a deep wound may affect the quality of the scar tissue. Thus, the mechanisms underlying the formation and maintenance of rete ridges have stimulated growing interest in the field of regenerative medicine. 

Preserving rete ridges or creating rete ridge-mimicking patterns is a subject of great concern in tissue engineering strategies to promote rapid and robust DEJ formation. Studies of rete ridges have been conducted after application of autologous epithelial cells as composite grafts with decellularized dermis in patients and in animal models [65,66,67,68]. The slow deposition of basement membrane proteins and the lack of rete ridges or dermal papillae result in poor epidermal-dermal adhesion and functional outcomes for patients treated with cultured epithelial autograft [69,70,71,72]. The combination of autologous cultured epithelial cells with a decellularized dermal matrix that retains the dermal papillae structure and basement membrane proteins has shown improvements in graft take, barrier function, and functionality [65,66,67]. 

The inclusion of rete ridges in engineered skin has been achieved by using scaffolds made from decellularized tissue [66,73,74,75]. Tissue engineering has generated skin equivalents that show resemblance in several ways to the native skin and mucosa, including partial replication of the rete ridge structure through microfabrication of a basement membrane analog in a bioengineered skin equivalent [76,77]. These studies revealed that the profiles of the rete ridge had influenced the stratification and differentiation of the epithelium. The development of materials that mimic the natural tissue architecture is thus a promising strategy. Plating keratinocytes on a microstructured collagen membrane mimicking the natural three-dimensional architecture of the human papillary dermis has revealed that a microfabricated dermal papilla template can direct keratinocyte behavior [78]. Keratinocytes cultured on microfabricated matrices with channels mimicking the native topographical microenvironment of the DEJ have demonstrated that keratinocyte differentiation increases as channel depth increases and channel width decreases. Furthermore, channels with the narrowest openings have enhanced epithelialization [77,79]. Adding fibroblasts to this model revealed that keratinocytes in narrower channels exhibit a more proliferative phenotype, whereas keratinocytes in wider channels exhibit enhanced synthesis of laminin-332 [80]. 

Electrospinning technology has attracted great interest in recent decades thanks to its easy and effective processing of a broad range of polymeric materials in the form of nanofibers. Collagen I–containing electrospun nanofibers have been reported to promote the adhesion and spreading of human epidermal keratinocytes and support the formation of epidermal layers [81]. Analysis of an electrospun polycaprolactone/collagen nanofibrous matrix coated with an ultrafine collagen I network in an in vitro wound gap model revealed a significant acceleration of keratinocyte migration over this matrix through the activation of β1 integrins, acquisition of a polarized phenotype, deposition of laminin-332, and expression of active matrix metalloproteinases (MMPs) [82]. Model material systems that support the growth of cultured human keratinocytes and mimic DEJ geography have been developed (feature diameter of 150 mm and center-to-center distance of 100 mm) and have revealed that topography is sufficient to direct the clustering of β1 integrin bright keratinocytes on top of the domed structures [42]. Further characterization identified a variable Rho kinase activity-dependent stiffness mechanism depending on the topography. Stem cells localized at the top displayed lower stiffness compared to those on the base, correlating with enhanced accumulation of E-cadherin, Desmoglein 3, and F-actin at cell-cell borders [83]. When a dynamic model was created in which the topography was applied after the cells were seeded, the clustering of β1 integrin bright keratinocytes occurred in the base of the undulations within 48 h, still in a Rho-kinase dependent manner [84]. It is likely that the mechanical characteristics of these topographically-patterned environments themselves have informed resident cells how to behave.

Other tissue-engineering strategies have been developed to provide a dermal platform with papillae-like structures. Collagen I films have been cast on corrugated polydimethylsiloxane molds and adhered to collagen sponges to create bi-layered skin with channel-like ridges, successfully resulting in an interdigitated DEJ, hosting the stem keratinocytes in its channel depths [80]. Laser processing of fibroblast-seeded electrospun scaffolds was then designed to produce engineered skin with an interdigitated interface allowing direct contact of keratinocytes and fibroblasts [85]. This micropatterned structure significantly increased the length of basement membrane, sped the development of epidermal barrier function, and increased the number of proliferating basal keratinocytes compared with a flat interface [85]. The combined use of autologous epithelial cells with a dermal template containing laser-micropatterned dermal papillae to treat full-thickness excisional wounds induced rapid and continuous basement membrane protein deposition as well as enhanced epidermal proliferation, differentiation, and stemness [86]. These important findings on the DEJ mechanics in the field of tissue engineering fit well with cell mechanobiology concepts (following paragraph) and recent findings in Drosophila melanogaster and Caenorhabditis elegans, two powerful in vivo models for basement membrane dynamics studies [87,88]. Altogether, these findings emphasize the role of basement membrane stiffness in shaping and maintaining the morphology of tissues and organs. 

Future studies will provide a better understanding on the impact of the topology of the DEJ on the biology of interfollicular stem cells as well as on the process of epidermal differentiation. A better understanding of the mechanisms will allow manufacturing materials suitable for optimal epidermal regeneration.

### 2.3. The Epidermis Is a Mechanosentive Tissue

Basement membrane components are tightly arranged into a thin, nanoporous layer. The nanometer-sized pores restrict cell movement and diffusion of very large molecules while permitting diffusion of smaller molecules. Basement membrane pore size is tissue-specific, and the DEJ is a porous and semipermeable filter that allows for the exchange of nutrients and fluids between the epidermal and dermal layers. The human DEJ contains numerous pores of 0.79 µm diameter situated beneath the junctions between keratinocytes [89]. No obvious relationship has been observed between the number of pores (28 × 10^3^ pores per mm^2^) in normal human skin and sex, age, or anatomical location [89]. The geometry of basement membrane structures makes measurement of their elastic properties difficult with current methods [90]. In addition, because of the challenges associated with their isolation and handling, knowledge is limited regarding the mechanical properties of native, human basement membranes. 

The skin undergoes constant mechanical stresses, including stretch and compression, because of body movement, touch, and growth of underlying tissues. Elasticity of mouse or human DEJ has not been reported, but AFM measurements have been made of the stiffness of some basement membranes. Young’s modulus is the mechanical property that measures the tensile stiffness of a solid material. In basement membrane derived from adult chick retina, values for this property were as high as 4.07 MPa [91], whereas those for moduli of corneal basement membrane ranged from 20 to 80 kPa [92]. AFM measurements have shown upper ranges for the stiffness of the basement membrane enveloping Drosophila eggs of 70 kPa [93] and 800 kPa [94]. This large variability in measurements of basement membrane stiffness likely arises from differences in membrane type and composition as well as in measurement techniques [95]. Models have been developed to specifically measure dermal-epidermal interaction strength in the context of diseases in which specific DEJ components are defective, such as EB. One group attempted to quantify the strength of the DEJ in a mouse model of EB by measuring the tension from the pull-push force gauge when a sleeve of tail skin was removed [96], and in vitro 3-dimensional (3D) useful models are currently being developed for quantitative assessment of the mechanical adhesion between the dermal and epidermal layers [97,98]. A biomechanical modeling of the DEJ undulating pattern was proposed, relying on the mechanical instability between dermal and epidermal layers and taking in account their microstructural properties and geometrical constraints [99,100]. Another model, which became more complex as the dermis/adipose tissue interface was taken into account, allowed for formulating new hypotheses concerning the impact of aging [101].

The ability of cells to sense and respond to ECM elasticity depends on their ability to deform it, a cell property that involves adhesion to the substrate and actin-myosin-mediated contraction against it. It is becoming increasingly evident that cell adhesion–mediated non-chemical signals such as mechanical forces and topography can be important in controlling epidermal cell behavior and fate. Although some signals such as mechanical forces may interact directly with intracellular components [102], many other signals are likely converted into intracellular chemical events where adhesion takes place. Adhesion-mediated non-chemical signals take a variety of forms. Applied mechanical forces can cause behavioral responses directly through intracellular signaling and indirectly through changes in gene expression [103,104]. Equally important extracellular signals include rigidity [105,106], shape [107,108], and topography [109]. 

In addition to a role in force bearing and force transducing, skin cells may actively sense the physical properties of their environment and respond by activating signaling cascades to control their fate and function. The epidermis is certainly a mechanosensitive tissue [110]. Although the DEJ structure and topography probably are the causes for a large portion of these signals, other causes are involved, as well. First, the skin has to expand during growth and development or during artificial tissue expansion, and data confirm that human epidermal tissues sense this increased tension through increased mitotic activity [111]. Studies of keratinocytes seeded onto a flexible surface subjected to various kinds of stretching have allowed deeper analysis of the pathways underlying this mechanical stimulation and supporting increased proliferation [112]. Early on, these studies identified integrin β1 and various signaling pathways, including the mitogen-activated protein kinase (MAPK), protein kinase C (PKC), and the ERK signaling pathways [113,114]. These findings provided support for the idea that physically stretched keratinocytes can sense their deformation by switching on intracellular signaling pathways.

Signaling pathways controlling cell shape and fate may arise from the geography of the microenvironment. When keratinocytes are constricted to small circular adhesive islands, they remain circular, differentiate more, and divide less than those grown on larger islands, where they fully spread. This pattern reveals that reduced adhesion and shape can act as signals for terminal differentiation [115]. Further studies using micro-patterned substrates established that simple changes in keratinocyte shape and adhesion are potent regulators of terminal differentiation [116]. That work revealed that when human epidermal stem cells attach to micropatterned substrates, the decision to differentiate does not depend on ECM concentration or composition or on integrin clustering in focal adhesions. Rather, it depends on the shape of the adhesive island so that for a given area, the proportion of cells that differentiate is higher on a circular substrate than on one that allows cell elongation. Cells with the highest stretching ratio maintained stemness to a greater degree [116]. Consistently, topographies that prevent cell spreading or restrict surface coverage promote differentiation through a contractility-dependent mechanism [117]. Although the precise mechanisms are unclear, these findings suggest that the 3D organization and topological features of the ECM play regulatory roles in mediating epidermal stem cell fate decisions. Coupling cell fate decisions with dynamic changes in ECM properties may allow cells to adjust their behavior to the changing needs of the tissue [118].

The stiffness of the ECM can have profound effects on the behavior of cells that interact with it. Cells grown in vitro on stiff collagen matrices activate Rho-associated kinase (ROCK) signaling, the principal driver of actomyosin contractility and cellular tension. In a process called “mechanoreciprocity” [119], this increased contractility generates counter-balancing cellular tension in an attempt to balance internal and external forces [120,121]. In murine epidermis, the conditional activation of ROCK results in significant modification of the ECM through increased collagen deposition, leading to increased tissue stiffness [122]. Accompanying the increased tissue stiffness, activation of the mechanically responsive β-catenin transcriptional co-activator promotes target gene transcription, and increased epidermal cell proliferation. Besides, the mechanical stretch stimulation of human skin equivalents was shown to induce epidermal thickening and DEJ protein synthesis and deposition [123]. These results show that cellular tension profoundly affects the external microenvironment and influences tissue homeostasis.

Keratinocytes seeded onto collagen or fibronectin-coated polyacrylamide (PA) of low elastic modulus (0.5 kPa) cannot form stable focal adhesions or spread and will undergo terminal differentiation within 24 h [124]. The keratinocytes differentiate as a result of decreased activation of the ERK/MAPK signaling pathway, which in turn reflects the failure of β1 integrins to cluster in focal adhesions. In addition, the reduced tethering of ECM molecules to cell culture supports induced terminal differentiation; when the ECM is loosely bound, it cannot provide the mechanical feedback that the integrin complex requires to cluster in focal adhesions and signal through ERK/MAPK. Thus, cells exert a mechanical force on substrate-bound ECM and gauge the feedback to make cell fate decisions [124]. The mechanisms underlying the control of these cellular events have been linked to the YAP/TAZ transcriptional network and mechano-control over the Notch signaling pathway, a key factor for epidermal differentiation [125]. Some authors have highlighted the lack of a substrate stiffness effect on MDCK cell proliferation [126], but others have found that proliferation, migration, and re-epithelialization of HaCaT cells are favored on a stiff substrate, and their differentiation reduced [127]. Their findings also indicated that a stiffer wound bed may be more favorable for rapid wound healing. Another study suggested a similar pattern by demonstrating that increased matrix stiffness promotes directional migration of HaCaT cells [128]. The mechanisms underlying HaCaT cell spreading and proliferation over a stiff support involve activation of the Wnt/beta-catenin and FAK-ERK pathways [129]. The use of silicone-based biomaterials with tunable mechanical properties revealed that elevated matrix stiffness beyond normal physiologic levels stimulates primary human keratinocyte proliferation in response to focal adhesion-dependent EGFR signaling pathway activation [130]. The softness-induced differentiation of keratinocytes was suggested to a reversible mechanism [131].

In contrast to previous findings, Zarkoob et al. found higher migration speed and colony-forming ability of human keratinocytes on soft PA gel surfaces (1.2 kPa) than on stiff PA surfaces (24 kPa) [132]. Keratinocytes on soft PA gels exhibited smaller spread contact areas and appeared to migrate directly towards an evolving multicellular aggregate in a cooperative manner, presumably in response to mechanical cues that propagated through the deforming substrate from the aggregate. By experimentally imposing substrate deformations similar in magnitude and rate of deformation to those that can be generated by an evolving multicellular aggregate of keratinocytes, the authors further revealed that isolated keratinocytes favored a course that vectorially aligned with the direction of substrate deformation, using a signaling pathway involving ROCK [132].

Collectively, these results provide compelling evidence supporting the notion that keratinocytes are mechanosensitive cells and that rigidity sensing of the DEJ can operate on multiple scales to affect keratinocyte behavior, including migration, proliferation, and differentiation.

## 3. Molecular Organization of the DEJ: Several Molecularly Interconnected Networks

The DEJ primary structural elements consist of two polymeric networks comprising laminin and collagen IV, which are primarily interconnected by nidogen and perlecan [3,14]. Biochemical and genetic characterizations in humans and animals have generated a basic model for how these components assemble [20,133,134,135]. Both collagen IV and laminin are trimeric proteins that self-assemble into independent networks. Laminin is regarded as the initiator of basement membrane assembly, whereas collagen IV, the most abundant basement membrane component, is responsible for its tensile strength. Recent studies of basement membrane repair mechanisms have suggested the possible existence of other modes of assembly. For example, during Drosophila larval epidermal repair, neither laminin nor collagen IV required other components to incorporate into the basement membrane, while perlecan depended on collagen IV for proper incorporation [136]. This genetically tractable system also revealed that the basement membrane proteins originated from the same cells as those involved in the *de novo* assembly mechanisms [136]. These results suggest that assembly mechanisms of the basement membrane during epidermal regeneration could differ from those occurring during development, as a tissue repair adaptation mechanism. 

**Figure 2:**
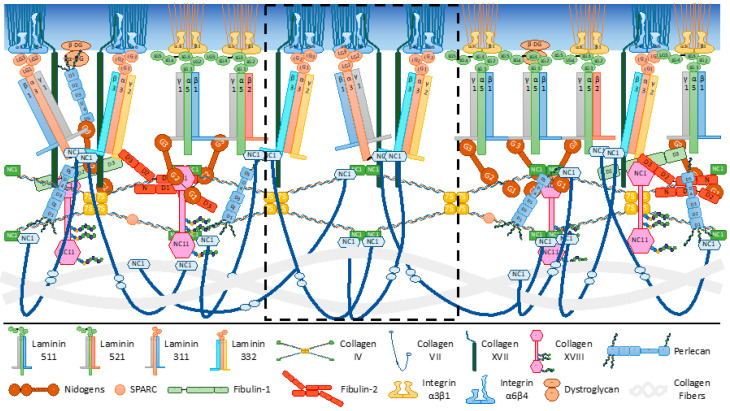
Dermal-epidermal junction components, receptors, and intermolecular binding. The epidermal basement membrane contains laminins, nidogens, collagens IV, VII, XVII, and XVIII, perlecan, and fibulins. Majors receptors and other cell surface binding molecules include integrins and dystroglycan. Collagen IV and laminins 511/521 form networks connected to one another by nidogens and perlecan. Other interacting partners and protein domains involved in molecular interactions are indicated. A focus on the extracellular matrix components of anchoring complexes and their interactions are presented in the central dotted frame. Laminin-332 and -311, collagen VII and XVII, and α6β4 integrin are shown. Laminins of the dermal-epidermal junction interact with keratinocytes receptors through the C-terminal laminin globular (LG) domains of their α chain. Non-collagenous (NC); globule (G), sedimentation coefficient 7S (7S), domain (D), N-terminal domain (N); β sub-unit of dystroglycan (β-DG); α sub-unit of dystroglycan (α-DG).

The collagen IV/laminin assembly pattern is reinforced in the DEJ by the anchoring complexes described above (Figure 2). Molecular organization of the DEJ in skin homeostasis is presented here, and the involvement of DEJ component in wound repair has been reviewed elsewhere [15,137].

### 3.1. The Archetypal Laminin/Collagen IV Networks

Early molecular studies as well as in vivo studies in Drosophila, C. elegans, and mice support the idea that laminin is the foundational building block for the initial formation of basement membranes [20,138]. All laminins are secreted heterotrimeric proteins consisting of three different gene products, the α, β and γ chains, assembled into an αβγ heterotrimer [139] (Figure 2). The principal domains include the laminin G-type (LG)1-LG5 modules at the carboxyl (C-) terminus of the α-chains, amino (N-) terminal globular domains (LN), and numerous laminin-type epidermal growth factor (EGF)-like (LE) modules that are related to EGF-like domains. Laminin trimers self-assemble into a polymeric sheet-like lattice that is tightly associated with the cell surface through their LG domains. Five α (α1, α2, α3, α4, α5), three β (β1, β2, β3), and three γ (γ1, γ2, γ3) subunits have been cloned, which assemble into the characteristic heterotrimeric structure [139,140,141,142]. Laminin networks are mainly non-covalent in nature and probably more dynamic than the collagen IV network. Most laminins can self-associate and form hexagonal networks [143,144]. This polymerization process is reversible and depends on both the concentration and the presence of divalent cations. This assembly model involves and absolutely requires the N-terminal domain LN of the α, β and γ subunits [145,146]. It therefore applies to laminins-511/521 (α5β1γ1/α5β2γ1) in the DEJ, the isoforms identified in this basement membrane, which contains the LN domain at its three N-terminal extremities [147,148] (Figure 2). Hence, collagen IV recruitment and further basement membrane assembly appear to depend on laminin self-assembly and its LG-mediated cell surface anchorage. 

Data from transgenic mice have revealed a role for laminin-511 in dermal–epidermal inter-communication and homeostasis [149,150]. In addition, laminin-511 is involved in mouse and human hair follicle morphogenesis by a mechanism supporting dermal hair papilla development and hair growth [151,152]. A mutation in the polymerization domain of the laminin α5 chain was recently identified in a patient with a complex developmental disorder affecting multiple organ systems [153]. A mouse model recapitulating this mutation revealed that the variant laminin is trimerized and secreted, suggesting that its failure to polymerize does not impede its incorporation into basement membranes [153]. However, no major skin defects were reported, and future studies will need to uncover the molecular status of the DEJ. From studies using organotypic cultures laminin 511/521 expression was shown to correlate with an increased ability of keratinocytes to regenerate rather than differentiate [154]. Further examination revealed that both keratinocytes and dermal pericytes secrete and deposited laminin 511/521 at the DEJ during this process [155,156].

Collagen IV, a ubiquitous component of all basement membranes, occurs as different isoforms depending on the chain composition of the molecules. Six different α1(IV) chains have been cloned and sequenced and exhibit tissue-specific expression [157]. The α1(IV) chains and α2(IV) chains are present in all basement membranes, including the DEJ, and most likely exert fundamental supportive functions given their highly cross-linked organization [134,158]. In the DEJ, both keratinocytes and dermal fibroblasts produce collagen IV [159,160,161]. Collagen IV forms intermolecular covalent bonds, a characteristic that gives the basement membrane its capacities to bear mechanical stress [162]. The collagen IV molecule [α1(IV)]2α2(IV) contains a C-terminal non-collagenous (NC) domain, NC1, and a relatively long triple-helical domain of 400 nm with several small interruptions allowing for a rather flexible helical rod and providing cell-binding sites. The molecules have the ability to self-aggregate into dimers by association of their NC1 domain and tetramers by association of their N-terminal domains [162,163,164] (Figure 2). The [α5(IV)]2α6(IV) molecule is expressed in the DEJ, but its precise function is unknown [165]. Work in Drosophila larvae has shown that collagen IV incorporation into basement membranes allows organ shaping through mechanical tensions [166].

### 3.2. The Interconnecting Molecules Central to the DEJ Integrity

Nidogen isoforms 1 and 2 are connecting elements between the laminin and collagen IV networks facilitating basement membrane formation and stabilization (Figure 2). The C-terminal G3 domain of nidogens links an EGF-like motif of the laminin γ1 chain [167], whereas its G2 N-terminal globule binds with high-affinity collagen IV [168]. Mice that lack both isoforms die shortly after birth from basement membrane abnormalities [169], but their skin has an ultrastructurally normal DEJ [170]. This pattern suggests that some basement membranes can form in vivo without nidogen or that other binding molecules such as perlecan may have compensatory and redundant functions.

Perlecan is an important proteoglycan of basement membranes and other ECM structures, and it consists of an elongated core protein modified by three or four HS chains. It has a multidomain structure and is composed of LG, LE, EGF-like, and immunoglobulin-like modules. Perlecan has a structural bridging function in the basement membrane and helps assemble the major constituents within molecular suprastructures [135,171,172] (Figure 2). As a proteoglycan, it acts as a reservoir for heparin-binding growth factors, limiting their diffusion and facilitating their ability to act on cells on either side of the basement membrane [173]. For instance, perlecan can influence keratinocyte survival and stemness [174,175]. In line with its function of basement membrane ultrastructural regulator, incorporation of perlecan within the collagen IV networks in the Drosophila larvae lessens constriction of the collagen IV scaffold, allowing the basement membrane to impact the tissue shape [166]. In addition to these important functions, HS proteoglycans were shown to regulate the hydration status of basement membranes, thereby determining their biomechanical properties, including thickness and stiffness [176,177].

Secreted protein acidic and rich in cysteine (SPARC) is a conserved matricellular and collagen-binding protein [178,179,180]. SPARC dysregulation perturbs the function of many ECMs, including basement membranes, and correlates with cancer progression [181,182]. SPARC (also known as BM40 or osteonectin) has been proposed to regulate growth factor activity, collagen deposition and degradation, and cell adhesion [183,184,185,186]. Recent work carried out in Drosophila and C. elegans development has uncovered essential elements for understanding the function of SPARC in regulating collagen IV deposition and its incorporation into the basement membrane, polymerization onto nascent laminin networks, and assembly [187,188,189,190] (Figure 2). 

Fibulins are multidomain proteins that also participate in diverse ECM supramolecular structures. All family members share tandem arrays of calcium-binding consensus sequences and have a diverse repertoire of interaction potentials, which makes them common components of the ECM. Fibulins-1 and -2 are found in the DEJ [191,192]. Nidogen-1, the C-terminal collagen XVIII fragment named endostatin, and the laminin γ2 chain are ligands of fibulins 1 and 2, whereas perlecan interacts with fibulin-2 [193]. Reduced fibulin-2 expression has been found in the DEJ of mice lacking integrin α3β1 in the epidermis, contributing to loss of basement membrane integrity and to skin blistering [194].

Collagen XVIII is a basement membrane-associated collagen that belongs to the multiplexin (multiple-helix domains with interruptions) subgroup within the collagen superfamily [195]. It is a homotrimer comprising three identical α1 chains [α1(XVIII)]3, and the non-collagenous C-terminal end contains the endostatin peptide, which can be released by proteolytic cleavage [195]. Collagen XVIII is expressed as three tissue-specific variants, and the so-called short isoform is dominant in vascular and epithelial basement membranes, including the DEJ [196,197]. Because of the three conserved serine-glycine consensus attachment sites for glycosaminoglycans, mainly of the HS type in humans, the short collagen XVIII is considered a proteoglycan [198]. Collagen XVIII exhibits a polarized orientation in which endostatin is embedded within the lamina densa and the N-terminal portion faces towards the basement membrane-fibrillar ECM interface [199,200] (Figure 2). A number of interactions of endostatin with basement membrane components have been identified [195], and in vivo studies have confirmed its anchorage to perlecan [201,202]. The ultrastructural data from knockout mouse models suggest that trimeric endostatin within full-length collagen XVIII binds to perlecan and other components to ensure the compact structure of the basement membrane [203]. A function of collagen XVIII in the regulation of the DEJ mechanical properties was revealed in 3D skin model [204].

### 3.3. The Distinctive and Essential Anchoring Complexes

Additional laminin isoforms are at the heart of anchoring complexes. Laminin-332 was discovered as the major component of anchoring filaments, where it mediates keratinocyte adhesion via interaction of its LG1-LG3 domains with both α3β1 and α6β4 integrins [9,12,205,206]. The α6β4 integrin initiates hemidesmosome formation by interacting with the cytoskeletal cross-linker plectin, which binds to cytoplasmic keratin intermediate filaments [207]. Basal keratinocytes assemble type I hemidesmosomes composed of integrin α6β4, plectin isoform 1a, bullous pemphigoid antigen1 isoform e (BPAG1e, also called BP230), bullous pemphigoid antigen 2 (BPAG2, also called BP180 or collagen XVII), and the tetraspanin CD151 [12,207,208], which are responsible for keratinocyte adherence, polarization, and spatial organization of tissue architecture. A recent examination using super-resolution microscopy has led to the definition of their molecular architecture [209]. The influence of cells on tissue mechanics is further illustrated by studies showing that the presence of α6β4 and its binding to keratin filaments via plectin and laminin-332 lowers the ability of keratinocytes to exert traction forces on the substratum [210].

Laminin-332 contains three subunits, α3, β3, and γ2. The α3 chain is also found in laminin-311, an isoform of composition α3β1γ1, found in association with laminin-332 in the DEJ [211]. To integrate the basement membrane, laminin-332 undergoes a proteolytic maturation process after secretion: both the α3 and γ2 chains are processed to shorter forms, whereas the β3 chain remains intact [18]. The γ2 chain cannot bind nidogen, and laminin-332, therefore, cannot associate with perlecan or the collagen IV network (Figure 2). Both α3 and γ2 lack the domain LN, which is known to promote laminin autoassembly. For this reason, laminin-332 is not found in this auto-assembled network. Networks containing laminin-332 have however been purified from skin, suggesting that some form of laminin assembly is possible [135]. Laminin-332 integration into the DEJ involves its cross-linking with laminin-311, which can bind nidogen through its γ1 subunit and append archetypal molecular networks [211] (Figure 2). The LG4-LG5 domains in the precursor α3 chain, shown to bind the HS receptors syndecans [212], generate small peptides involved in wound healing and host defense [18,213].

Another mechanism is based on the direct binding of anchoring filaments to anchoring fibrils. Anchoring fibrils are disulfide bond-stabilized dimers of collagen VII [214,215] (Figure 2). They are centrosymetrically cross-banded, fibrillar structures that originate at the lamina densa and extend into the dermis, looping into the upper regions of the papillary dermis and reinserting into the lamina densa. The loops surround and entrap the fibrous dermal elements, securing the basement membrane to the dermis. Collagen VII is a nonfibrillar collagen composed of three identical α1 chains [α1(XVIII)]3. The initially synthesized polypeptide of collagen VII, the α1(VII) chain, contains a large N-terminal globular domain, NC1, an unusually long and interrupted triple-helical domain (450 nm), and a relatively small C-terminal globular domain, NC2. The NC2 domain, believed to facilitate the formation of the antiparallel dimers, is proteolytically removed during chain maturation of the anchoring fibrils, and the NC1 domain is formed by the assembly of the three α1(VII) that are separately folded [216]. The dimers are covalently cross-linked through disulfide bonds at the carboxy terminus, and they aggregate laterally to form the anchoring fibrils. Collagen VII binds to laminin-332 and collagen IV through the NC-1 domain in the epidermal basement membrane zone and to collagen I in the dermis [11,217]. Monomeric laminin-332 and the laminin-332/311 dimer directly bind the N-terminal NC1 domain of collagen VII [217,218]. The interaction is likely to occur within the LN domain of the β3 chain and the fibronectin-like III repeats within the NCI domain of collagen VII [219,220]. 

The laminin β3 chain also interacts with the extracellular collagenous domain of collagen XVII [221,222]. Collagen XVII is a type II transmembrane collagen homotrimer of three α1(XVII) chains, each with a globular cytosolic N-terminal domain, a short transmembrane stretch, and a flexible-rod extracellular C-terminal domain [223]. The extracellular domain extends into the lamina densa and loops back into the lamina lucida, and its cytoplasmic tail binds to integrin α6β4, BP230, and plectin [12,224]. Staining for non-hemidesmosomal collagen XVII has been found at the intercellular spaces between basal keratinocytes [225]. Thus, collagen XVII is thought to be a vital cell surface receptor that links the cytoplasmic structural components with the ECM.

The importance of the anchoring complexes in dermal-epidermal cohesion is illustrated in both inherited and acquired blistering diseases. Congenital inherited EB comprises a group of disorders characterized by mutations in any of the genes encoding the structural components of the anchoring complexes [226,227,228,229,230]. The breakage can occur within the epidermis, along the DEJ, or in the upper dermis. Four main types of EB have been described, based on the morphological level of separation within the DEJ zone: EB simplex, junctional EB, dystrophic EB, and Kindler syndrome [226]. A common hallmark of all EB types is trauma-induced skin blistering and fragility, but each type also encompasses a number of subtypes, among which the extent of skin lesions and the associated organ manifestations can vary substantially. At least 20 genes may be involved in EB, and secondary phenomena, such as inflammation or fibrosis, can worsen the disease. No cure is yet available, but novel approaches to curing the disease and alleviating its symptoms are currently in development [230,231].

Several molecules of the DEJ have been identified as autoantigens in a heterogeneous group of disorders known as pemphigoid diseases [232]. Binding of autoantibodies to their target antigens leads to separation of the epidermis and dermis. The molecular identification of these proteins has led to the development of different serological test systems that facilitate the differential diagnosis of these disorders [232,233].

## 4. Tense Environment at the DEJ during Skin Aging

### 4.1. Flattening of Epidermal Rete Ridges

**Figure 3:**
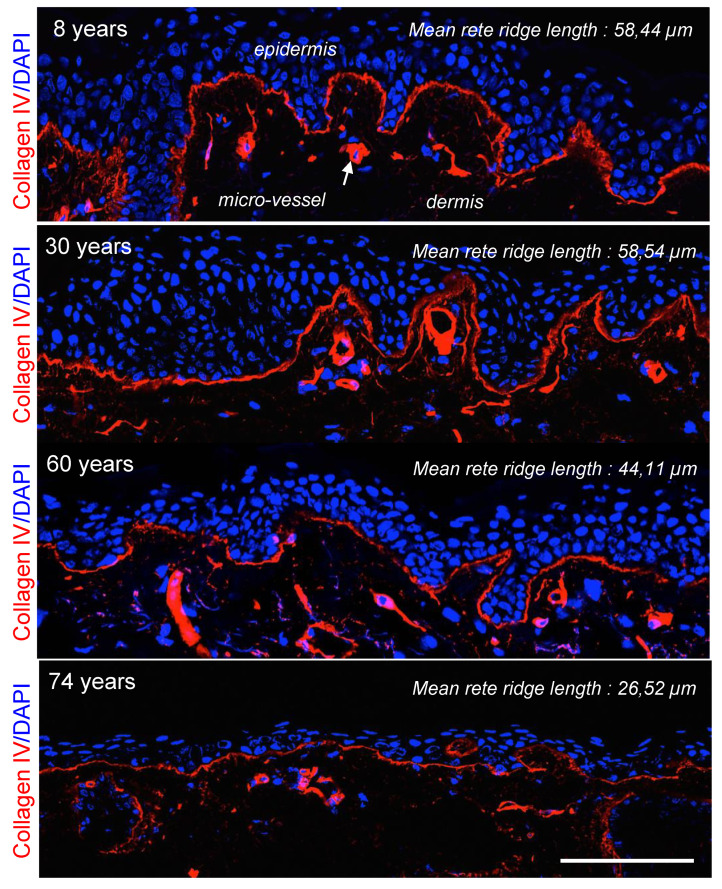
Collagen IV expression in facial human skin from female donors aged 8, 30, 60 and 74 years. Sections of frozen Tissue-Tek^®^ O.C.T.™-embedded skin biopsies were stained with the monoclonal anti-collagen IV antibody (Clone 94, Sigma-Aldrich, St. Quentin Fallavier, France) and the nuclei were stained with DAPI. Note the regular lessening of collagen IV labeling at the dermal-epidermal junction and the gradual disappearance of rete ridges with the increase in the age of donors. Epidermis, dermis, micro-vessels and the mean rete ridge length (*n* = 10) are indicated. Scale bar = 100 μm.

Studies have shown histologically that a consistent feature of aged skin is flattening of the DEJ by approximately 35%, with progressive loss of rete ridges and dermal papillary projections [45,234,235,236,237]. The rete ridge height decreases with age [30,32] (Table 1, Figure 3), and the number of papillae per area decreases with time [238,239]. The changes observed during aging vary according to the body sites: dermal papillae are uniformly distributed in the photo-protected abdominal skin, but they appear to be severely decreased and non-uniformly distributed in the facial skin [47] (Figure 3).

The interdigitation index, a two-dimensional measurement of the interdigitation in the DEJ, is also reported to decrease with age, by about 20% from young to old age [240]. Using in vivo non-invasive microscopy techniques such as reflectance confocal microscopy or second- and third-harmonic generation microscopy, studies have demonstrated that the flattening of epidermal ridges and dermal papilla pattern occurs progressively with time [28,47,241,242]. The average height of the dermal papillae as well as the contact area between the epidermis and dermis are therefore negatively correlated with aging [241].

The changes in the dermal-epidermal interface in aged skin have significant physiologic consequences. The epidermis is firmly attached to the dermis at the DEJ to provide resistance against mechanical shearing forces, and recent findings emphasize the impact of basement membranes on the mechanical properties of tissues [90,243]. A relatively flat DEJ results in a decreased contact surface area between the epidermis and dermis, which leads to skin fragility and weakens the exchange of nutrients, oxygen, and waste products between these two layers. This pattern is in accordance with the clinical observation that aged skin is prone to friction trauma and has compromised wound-healing capacities [244].

Although the process of changes in appearance and flattening of rete ridges during aging has been well demonstrated, the underlying causes are still unknown. The flattening process may result from a reduction in dermal papilla height [45,245], as part of the papillary dermis. Intrinsic skin aging involves a number of dermal changes, including dermal atrophy, decreased collagen biogenesis, and loss of normal elastic fibers [246,247,248]. Aging strongly affects papillary fibroblasts, which undergo a number of genomic and morphologic changes that gradually transform them into reticular-like fibroblasts [249,250,251,252]. The seeding of papillary fibroblasts in the dermal compartment of in vitro 3D reconstructed skin samples promoted the formation of a well stratified and differentiated epidermis, while those seeded with reticular fibroblasts did not [249]. When these models were grafted into nude mice, those containing papillary fibroblasts isolated from the skin of young donors formed well delineated and regular rete ridge-like structures while those containing reticular or older papillary fibroblasts did not [249]. These data strengthen the importance of the dermal-epidermal crosstalk in the DEJ characteristics and highlight its failure during aging. ECM produced by and surrounding papillary dermis fibroblasts undergoes profound alterations during skin aging. The curled, thin, short, and loosely interwoven fibers become thicker and form network aggregates [253,254,255,256], leading to assessable changes in the overall tissue stiffness [257]. These changes are more severe in photo-exposed skin [242,254]. UV irradiation induces high levels of MMP expression, and these proteins are responsible for degradation of collagen fibers, elastic fibers, and fibrillin microfibrils, as well as defects in transforming growth factor (TGF)-β pathway activation [258,259,260]. Another cause of aging-associated DEJ flattening may result from the shrinkage and sagging of the terminal elastic fiber arcades, which are distributed in the papillary dermis and extend to the epidermis in the skin of young people [45].

The disappearance of rete ridges might also involve an epidermal contribution. Studies have shown a statistically significant decrease in the human epidermal stem cell markers β1 integrin and melanoma chondroitin sulfate proteoglycan in the basal layer of aged epidermis, leading to a reduction in basal cell density and possibly affecting the height of the rete ridges [32].

### 4.2. Deterioration of the DEJ Molecular Scaffold during Aging

Among other basement membranes, an age-dependent increase in DEJ thickness has been reported in humans [261]. AFM measurements have revealed an age-related increase in human basement membrane stiffness [176], suggesting that basement membranes from humans with advanced age may become thicker and stiffer [21].

A large number of studies have reported that most DEJ components are altered during aging. Immunolabeling analysis revealed reduced expression levels of laminin-332, integrin β4, collagen IV, collagen VII, collagen XVII, and collagen XVIII in the skin of older people [57,204,261,262,263,264]. A clear decreased expression of epidermal perlecan was revealed during skin aging at both protein and mRNA levels [175,265]. Exogenous human perlecan was shown to recover the thin epidermal layer obtained with aged keratinocytes in a 3D skin equivalent model [175]. After reaching the DEJ, the added perlecan allowed the development of a well-differentiated multilayered epidermis displaying increased expression of Ki67 and keratin 15 in the basal layer [175]. Collagen XVII expression is highly reduced during aging, causing hemidesmosome alteration and leading to keratinocyte detachment from the DEJ and upward migration [266]. In addition to its important function in maintaining dermal-epidermal cohesion [225], collagen XVII has a role in the proliferation of interfollicular keratinocytes and morphogenesis of hair follicles. All of these functions can be affected by its deficiency during aging [267,268]. Analysis in mice has shown that stem cells expressing a high level of collagen XVII have higher stemness potential and are thus selected for epidermal homeostasis [266]. Their loss of collagen XVII expression limits this potential, consequently causing aging. Another study, focused on hair follicle stem cells during aging, identified that collagen XVII depletion in hair follicle stem cells is a key factor to hair follicle shrinkage and hair loss [269].

TGF-β receptor expression is reduced in senescent fibroblasts, leading to reduced collagen IV synthesis [264]. In addition, collagen IV undergoes extensive proteolysis by MMP-10 and -7, compromising the stability of its numerous molecular interactions [270]. Age-related TGF-β signaling depletion also affects collagen VII expression, directly weakening the epidermal anchoring structures [271]. The defect in collagen VII expression worsens in photo-exposed skin because of the proteolytic activity of MMP-8 and -13 [270,272]. Age-related changes and environmental stress cause alterations in the balance between degradation and synthesis of proteins. The overexpression and activation of MMPs during aging not only can degrade collagen and elastin fibers in the dermis but also target DEJ components, disturbing their mechanical properties and affecting cell behavior through the regulation of microenvironment stiffness [273]. UV-exposure induces expression and activity of MMP-1, -3, and -9, resulting in the degradation of laminins, collagen IV, VII, and XVIII, nidogen, and perlecan [270,274]. The appearance of senescent cells in skin during chronological aging is also a source of MMPs that are prone to degrading DEJ components [275,276,277]. These events induce age-related damage and ultrastructural changes, which are all the more exacerbated in photo-aged skin [13,278,279]. MMPs also can regulate the expression and activity of cytokines, chemokines, and growth factors, in turn affecting the regulation of stem cell behavior. For example, the enhanced activity of heparanase in sun-exposed skin triggers severe degradation of HS chains. Consequently, growth factors are released, causing many alterations in skin homeostasis, such as keratinocyte hyperproliferation [280]. The reduction in perlecan dampens the release of growth factors such as the heparin-binding EGF, depriving the keratin 15-expressing stem cells in aged skin, impacting the epidermis renewal capacity [175].

Altogether, changes of the ECM composition and structure progressively modify the mechanical integrity of the DEJ and lead to an impaired response of cells and tissues to mechanical forces [130,281]. For example, reduced expression of collagen XVIII in the DEJ results in decreased elasticity and strength of human skin [204]. As documented in paragraph 2.3, such mechano-dependent control of epidermal cells may have significant consequences on the fate of progenitor cells during ageing [115,116,124,282,283]. Modification in DEJ organization and stiffness affects the stem cell niche, resulting in alteration and depletion of stem cell numbers. Laminin-332 is highly expressed by CD271-expressing interfollicular stem cells at the tip of dermal papillae, influencing their proper capacity to differentiate through the Notch signaling pathway and promote epidermal turnover [57]. The loss of laminin-332 expression in the stem cell niche during aging may cause an impairment of the epidermal differentiation process [57]. As laminins are believed to play a major role in basement membrane stability [21], whether the loss of laminin-332 during skin aging impacts ECM stiffness is an attractive hypothesis to explore.

Changes in the mechanical properties of cells are, as well, hallmarks of the aging process [284,285]. Because of the constant renewal activity of the epidermis, the age-related changes in intrinsic ability of epidermal cells to sense and transduce mechanical signals may strongly affect DEJ composition and shape [286,287]. AFM studies of epithelial cells, including keratinocytes, have revealed that cell cytoplasmic compliance or deformability is reduced with age [288,289]. This age-dependent stiffening affects the cell edge, cytoplasm, and perinuclear region [288]. A model reproducing undulations in the DEJ revealed that epidermal stem cell patterning appears to depend on mechanical forces exerted at intercellular junctions in response to these undulations [83].

ECM components also can be affected by non-enzymatic post-translational modifications during aging. The best-known target is the glycation process known as the Maillard reaction, in which a reactive carbonyl group of a reducing sugar such as glucose reacts with a free amino group of a protein, generating advanced glycation end products (AGEs). AGEs are produced endogenously at low rates during metabolic processes but can increase in number because of exogenous factors such as diet, smoking, or UV irradiation [290]. In skin, glycation affects cells (endothelial cells, fibroblasts, keratinocytes) and structural proteins such as collagen, elastin, glycoproteins, and glycosaminoglycans. Glycation modification of the dermal ECM further affects growth, differentiation, and motility of fibroblasts, the cytokine response, enzymatic activity, and vascular hemostasis [291]. The presence of AGE in the dermal ECM leads to intermolecular cross-linking of collagen and elastin, triggering many structural modifications that might affect potential binding sites for other components or cells, as well as inducing tissue stiffening and loss of elasticity [292]. Because collagen and elastin fiber turnover is slow, AGE-rich collagen and elastin molecules accumulate in the skin during aging [293].

Basement membrane components are also targets for glycation, and the DEJ is no exception. In vitro experiments have revealed that glycation of collagen IV and laminin alters their structure, ability to polymerize, and capacity to promote cell adhesion [294]. Such studies were subsequently carried out with endothelial cells and demonstrated the deleterious impact of collagen IV and laminin glycation on both the cellular and molecular interactions of these two major basement membrane components [295]. Studies of the glomerular basement membrane have revealed that glycation of collagen IV in the triple helix domain greatly inhibits its turnover and release [296]. UV radiation may amplify the phenomenon in skin by further reducing keratinocyte expression of lysosomal/endosomal cathepsins, which are proteases involved in ECM protein turnover, resulting in an abnormal deposition/turnover of DEJ proteins and accumulation of altered proteins [297]. Defects in collagen IV and VII expression have been found at the DEJ of a reconstructed skin model made of a dermal compartment prepared with preglycated collagen [298]. Some consequences of glycated dermal collagen can be seen in the epidermis where alterations in integrin expression, and in the differentiation process, have been reported [298,299].

AGEs exert their deleterious actions not only because of their biological properties per se but also through their interaction with specific receptors. Receptor for AGEs (RAGE) is a multiligand member of the immunoglobulin superfamily of cell surface receptors. The binding of ligands to RAGE stimulates various signaling pathways, including the MAPK, ERKs 1 and 2, phosphatidyl-inositol 3 kinase, p21Ras, stress-activated protein kinase/c-Jun-N- terminal kinase, and the janus kinases [300,301]. Stimulation of RAGE results in activation of the transcription factor nuclear factor kappa-B and subsequent transcription of many proinflammatory genes. In the skin, RAGE expression has been observed in both epidermis and dermis and is increased in sun-exposed compared with UV irradiation-protected areas. Keratinocytes, fibroblasts, and dendritic cells express RAGE, as do endothelial cells and lymphocytes to a lesser extent [302]. In keratinocytes, RAGE decreases cell proliferation, induces apoptosis, and increases MMP production [303].

All of the cellular and extracellular alterations lead to structural and functional anomalies and generate skin fragility and dysfunctions. Dermatoporosis describes the different manifestations of extreme cutaneous fragility apparent in the skin of aged people [304]. The DEJ is at the heart of communication between the dermis and epidermis and performs a central biomechanical function both in maintaining epidermal homeostasis and during the regeneration process. As a consequence of aging, the skin becomes more fragile, less resistant to shearing forces, and more vulnerable to injury, which, in addition to other compounding age-associated factors, leads to a much higher incidence of chronic wounds in the elderly [305]. The causes of age-related DEJ dysfunctions are multiple, and it appears clear now that the stiffness of the ECM can have profound effects on the behavior of the cells that interact with it. The recent findings showing the biomechanical properties of basement membranes open new and interesting perspectives, leading to promising research avenues. Understanding how biophysical cues are transduced into transcriptional responses, thus determining the fate of epidermal cells, will allow for identifying their dysfunction during aging.

## Figures and Tables

**Table 1:** Length of epidermal rete-ridges in human skin over age in various anatomical locations.

Age of Donors	Cohort	Anatomic Location	Mean Length (µm)	Technology Used	Reference
3 months	7 female, 8 male	Upper thigh	50 (estimated)	in vivo Confocal Laser Scanning Microscopy	[29]
		Ventral forearm	35 (estimated)		
		Buttock	48 (estimated)		
18–30 years	6 female, 4 male Fitzpatrick skin phototype I-III	Forearm	37.6 ± 6.9	Immunostaining on paraffin section and imaging measurements	[28]
		Buttock	61.7 ± 13.2		
19–24 years	8 female, 7 male Caucasian	Face–Temple	30 ± 8	in vivo Confocal Laser Scanning Microscopy	[30]
		Forearm	41 ± 8		
19–29 years	5 female, 7 male Caucasian	Volar arm	97.41 ± 25.93	in vivo Harmonic Generation Microscopy	[31]
21–33 years	10 (mix) Caucasian	Abdomen	60 (estimated)	Immunostaining on frozen sections and imaging measurements	[32]
30–59 years	5 female, 8 male Caucasian	Volar arm	69.59 ± 23.96	in vivo Harmonic Generation Microscopy	[31]
40–65 years	41 female French Fitzpatrick skin phototype II–IV	Forearm	27.01 ± 12.73	in vivo Reflectance Confocal Microscopy	[33]
		Face	13.66 ± 12.73		
40–65 years	41 female Brazilian Fitzpatrick skin phototype II–IV	Forearm	28.16 ± 8.23		
		Face	9.21 ± 12.41		
51–59 years	7 (mix) Caucasian	Abdomen	35 (estimated)	Immunostaining on frozen sections and imaging measurements	[32]
54–57 years	5 female, 10 male Caucasian	Face–Temple	20 ± 6	in vivo Confocal Laser Scanning Microscopy	[30]
		Forearm	25 ± 8		
>60 years	6 (mix) Caucasian	Abdomen	15 (estimated)	Immunostaining on frozen sections and manual measurements	[32]
60–79 years	2 female, 4 male Caucasian	Volar arm	58.97 ± 16.70	in vivo Harmonic Generation Microscopy	[31]
>65 years	6 female, 4 male Fitzpatrick skin phototype I-III	Forearm	26.2 ± 4.5	Immunostaining on paraffin sections and manual measurements	[28]
		Buttock	46.0 ± 15.3		
74–81 years	209 participants Fitzpatrick skin phototype I–IV	Face	5.98 ± 7.11	in vivo Reflectance Confocal Microscopy	[34]
		Forearm	10.55 ± 8.01		
		Volar arm	12.94 ± 5.42		
	105 females Fitzpatrick skin phototype I–IV	Face	6.41 ± 7.08		
		Forearm	10.00 ± 8.49		
		Volar arm	12.37 ± 5.02		
	104 males Fitzpatrick skin phototype I–IV	Face	5.57 ± 7.13		
		Forearm	11.06 ± 7.54		
		Volar arm	13.50 ± 5.75		
